# Supplementary material for: Evolution of the SARS-CoV-2 Mutational Spectrum
Source: Mol Biol Evol. 2023 Apr 11;40(4):msad085. doi: 10.1093/molbev/msad085 (PMC10124870; doi:10.1093/molbev/msad085)
Supplement: msad085_Supplementary_Data [file msad085_supplementary_data.pdf]

Supplementary Material

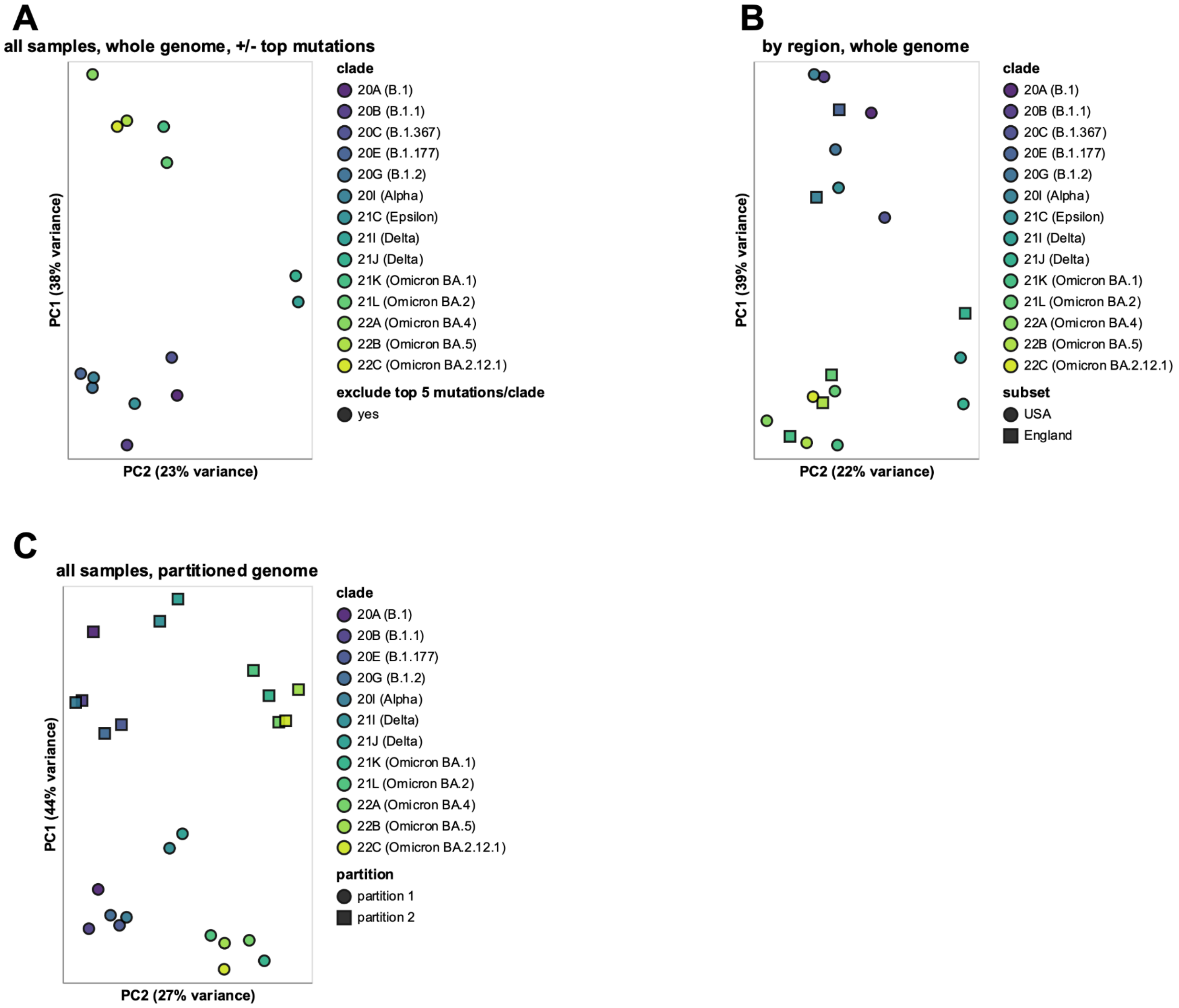

Figure S1. The inter-clade differences in mutation spectrum are robust to various possible sources of noise. This figure repeats the PCA in Figure S1 and shows that the results are robust to (A) excluding sites of the top-5 most abundant mutations in each clade, (B) examining only sequences from the USA or England, or (C) partitioning the genome into half. For panel C, some structure in the PCA plot is explainable by the genome partitioning but the shift in points caused by partitioning the genome is consistent across all clades, and so is not responsible for the inter-clade differences. These plots can be more easily explored using the interactive versions at https://jbloomlab.github.io/SARS2-mut-spectrum/ that enable mousing over of points and clicking on the legend to choose specific clades or groupings.

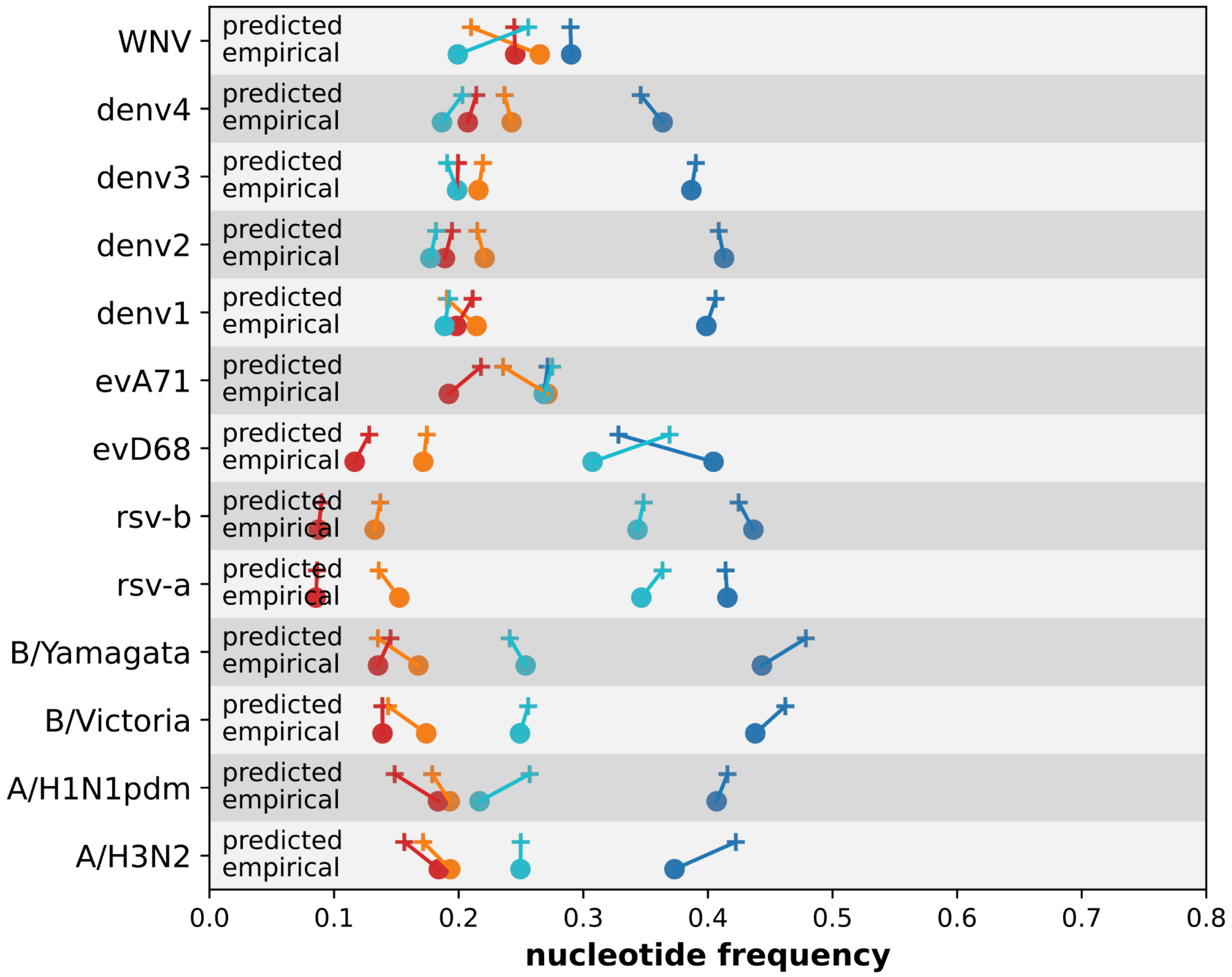

Figure S2. Predicted versus empirical nucleotide frequencies for all of the non-SARS-CoV-2 viruses shown in Figure 3C. The empirical frequencies just represent the nucleotide identities at four-fold degenerate sites, and the predicted frequencies represent the nucleotide frequencies expected based on the mutation rates estimated for these four-fold degenerate sites.

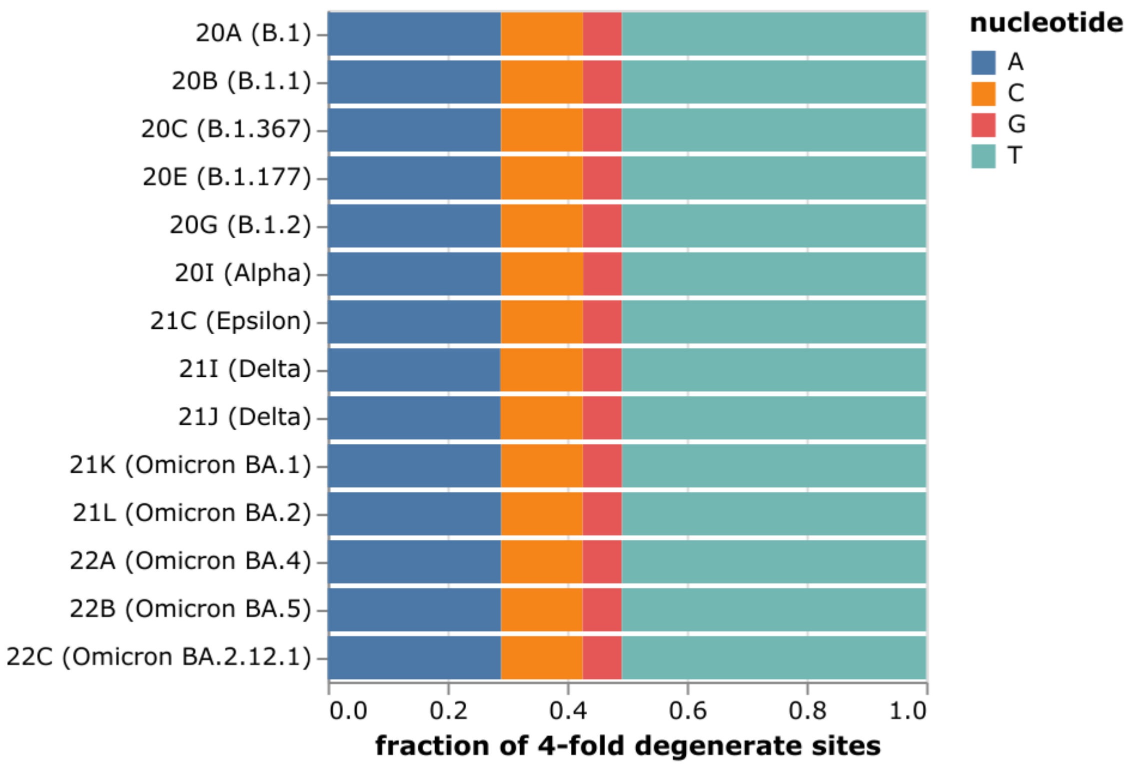

Figure S3: The frequencies of nucleotides at four-fold degenerate sites are nearly identical among the clade founder sequences. Note the high similarity among nucleotide frequencies at these sites is unsurprising as the pairwise nucleotide identity of current SARS-CoV-2 variants is very high (e.g., 99.8% full-genome nucleotide identity between Wuhan-Hu-1 and Omicron BA.5).
